# Case Report: First-Line Immunotherapy for Esophageal Squamous Carcinoma Combined With Hypopharyngeal Squamous Carcinoma Yields Sustained Survival Benefit

**DOI:** 10.3389/fimmu.2022.907705

**Published:** 2022-07-11

**Authors:** Yi Yang, Xiangliang Liu, Wei Song, Jin Lu, Na Yin, Xiaojun Ye, Xiao Chen

**Affiliations:** Cancer Center of the First Hospital, Jilin University, Changchun, China

**Keywords:** esophageal cancer, chemotherapy, immunotherapy, sustained survival benefit, hypopharyngeal squamous cell carcinoma

## Abstract

Esophageal cancer, as one of the most common malignant tumors in the upper gastrointestinal tract, is highly invasive, with poor prognosis and low 5-year survival rate. Hypopharyngeal cancer has a low incidence among head and neck malignant tumors, but its prognosis is poor and it is prone to recurrence, and because the upper respiratory tract has similar tissue types as the upper gastrointestinal tract, it is prone to the second primary tumor of the upper gastrointestinal tract, however, such patients with double primary carcinoma are uncommon in the clinic, and most of them are already advanced at the time of diagnosis, losing the chance of surgical resection, with poor results and poor prognosis after radiotherapy treatment, therefore, the choice of treatment strategy for such inoperable resectable patients is still a great challenge for clinicians.In this case, we report a patient with a double primary esophageal squamous carcinoma combined with hypopharyngeal squamous carcinoma without family history of tumor, who achieved complete remission after first-line chemotherapy combined with immunotherapy, with both lesions shrinking and the hypopharyngeal tumor disappearing. The survival benefit was ensured at the same time.

## Case Report

**Figure 1:**
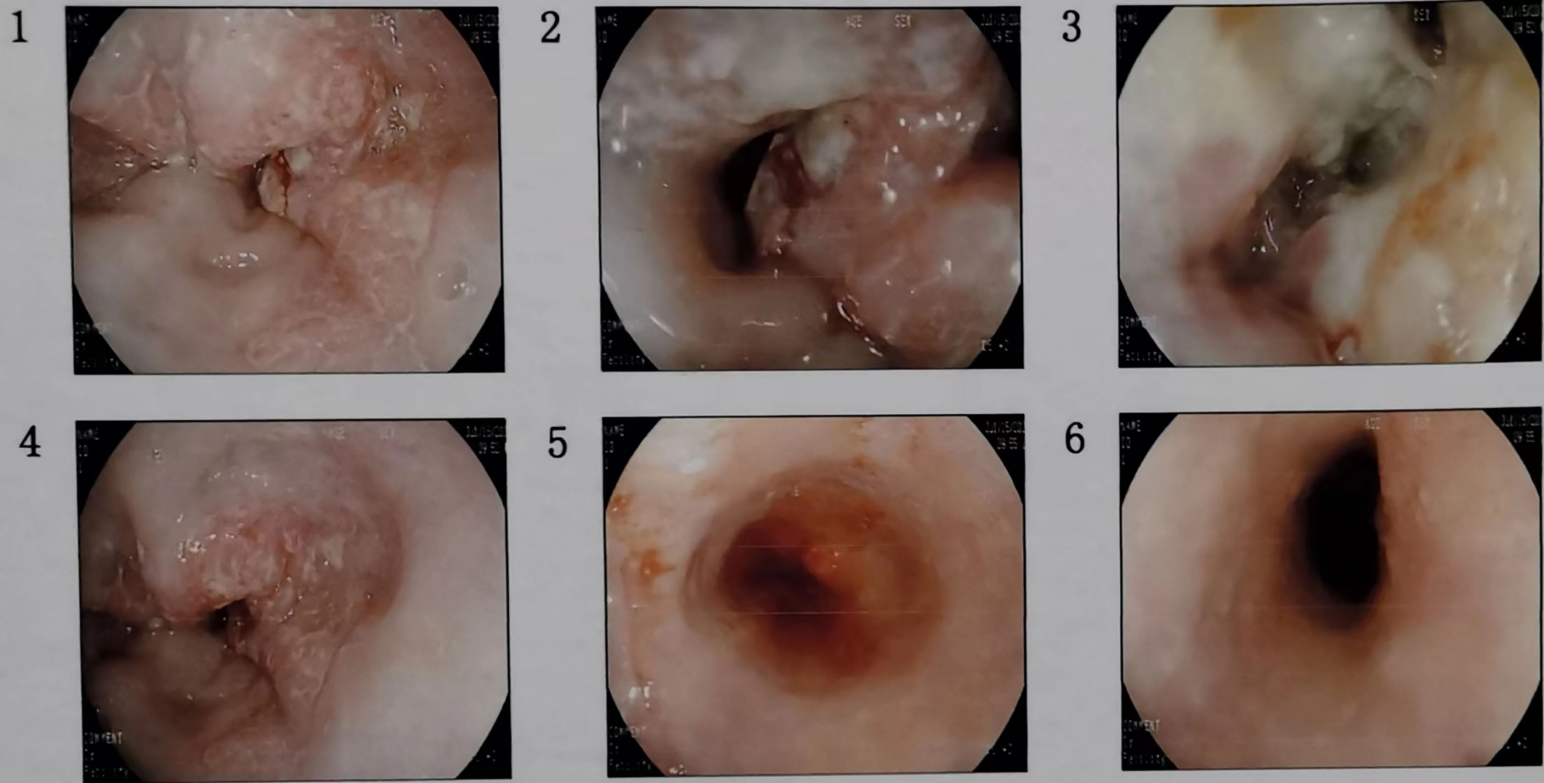
Consecutive gastroscopy images showed an irregular ulcer-type tumor of about 3.0x4.0cm in size at the esophagus about 32cm away from the incisors, the surrounding mucosa was raised like a bank, the bottom was covered with dirty moss, the surface was uneven, the tissue was brittle, it was easy to bleed, and the lumen was deformed and narrowed.

**Figure 2:**
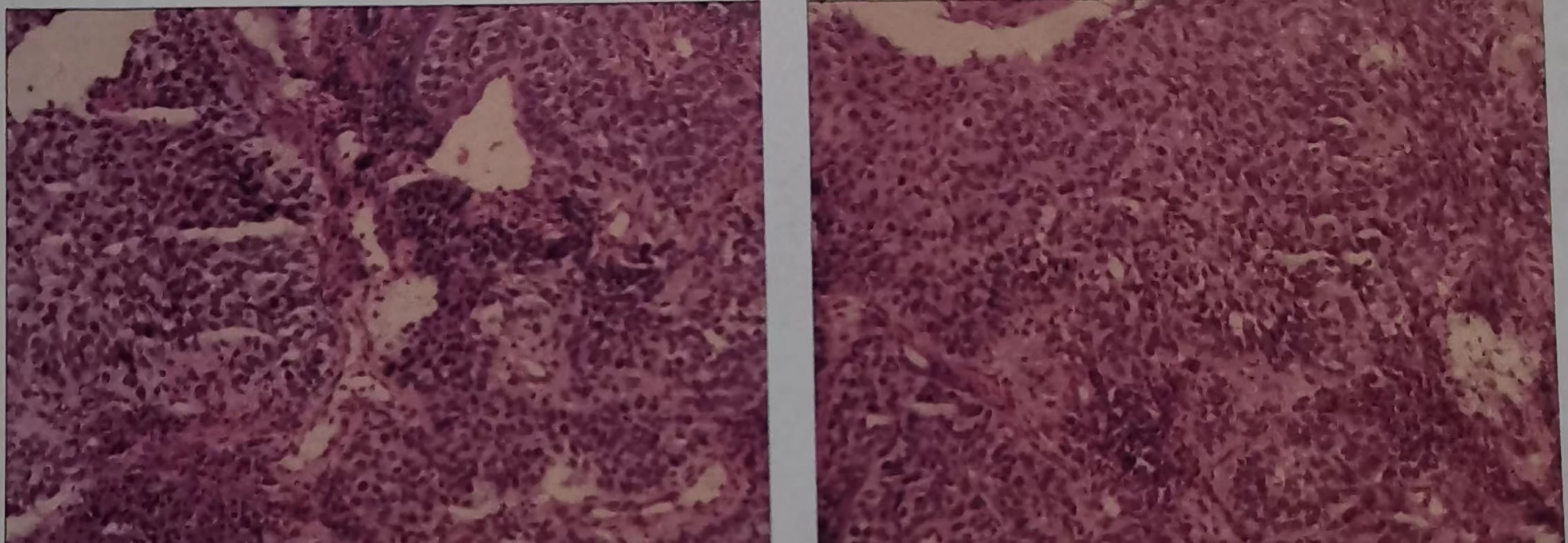
Haematoxylin and eosin-stained sections of low-differentiated squamous cell carcinoma in the esophagus (ESCC). (On July 16, 2020, tissue biopsy specimens were taken from the lower esophagus under gastroscopy.).

**Figure 3:**
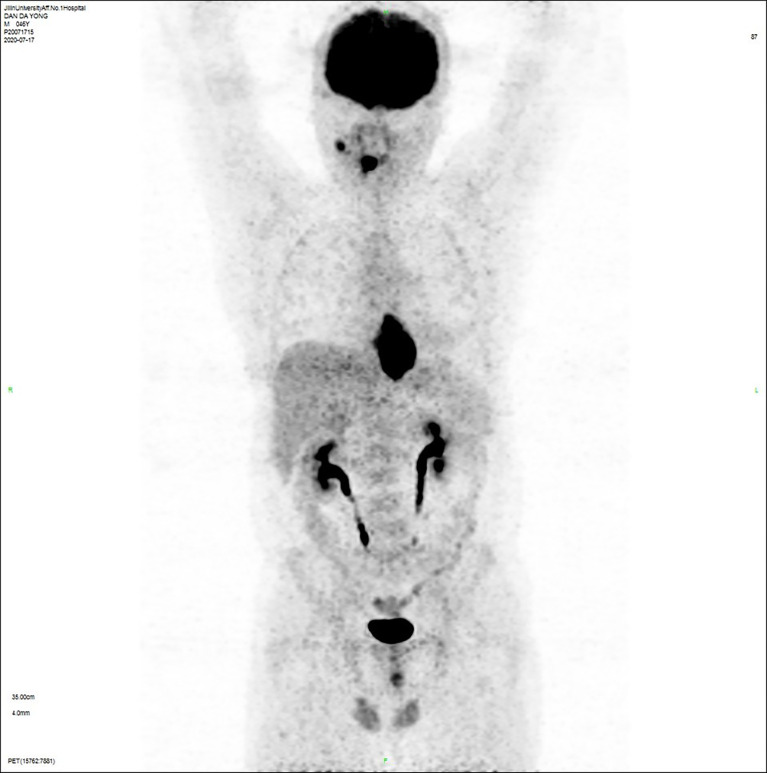
Positron emission tomography/computed tomography (PET/CT) examination showed that: **(a)** the right hypopharyngeal wall was thickened with increased metabolism, hypopharyngeal cancer was considered, **(b)** the esophageal wall was limited to hypermetabolic foci at the level of the thoracic 6-10 vertebral body, which was considered esophageal carcinoma, infiltrated to the plasma membrane layer and involved the gastric cardia, **(c)** the right submandibular hypermetabolic lymph node, the small lymph nodes adjacent to the lower esophagus and the gastric cardia had slightly higher metabolism, which were all considered to be metastatic cancer.

**Figure 4:**
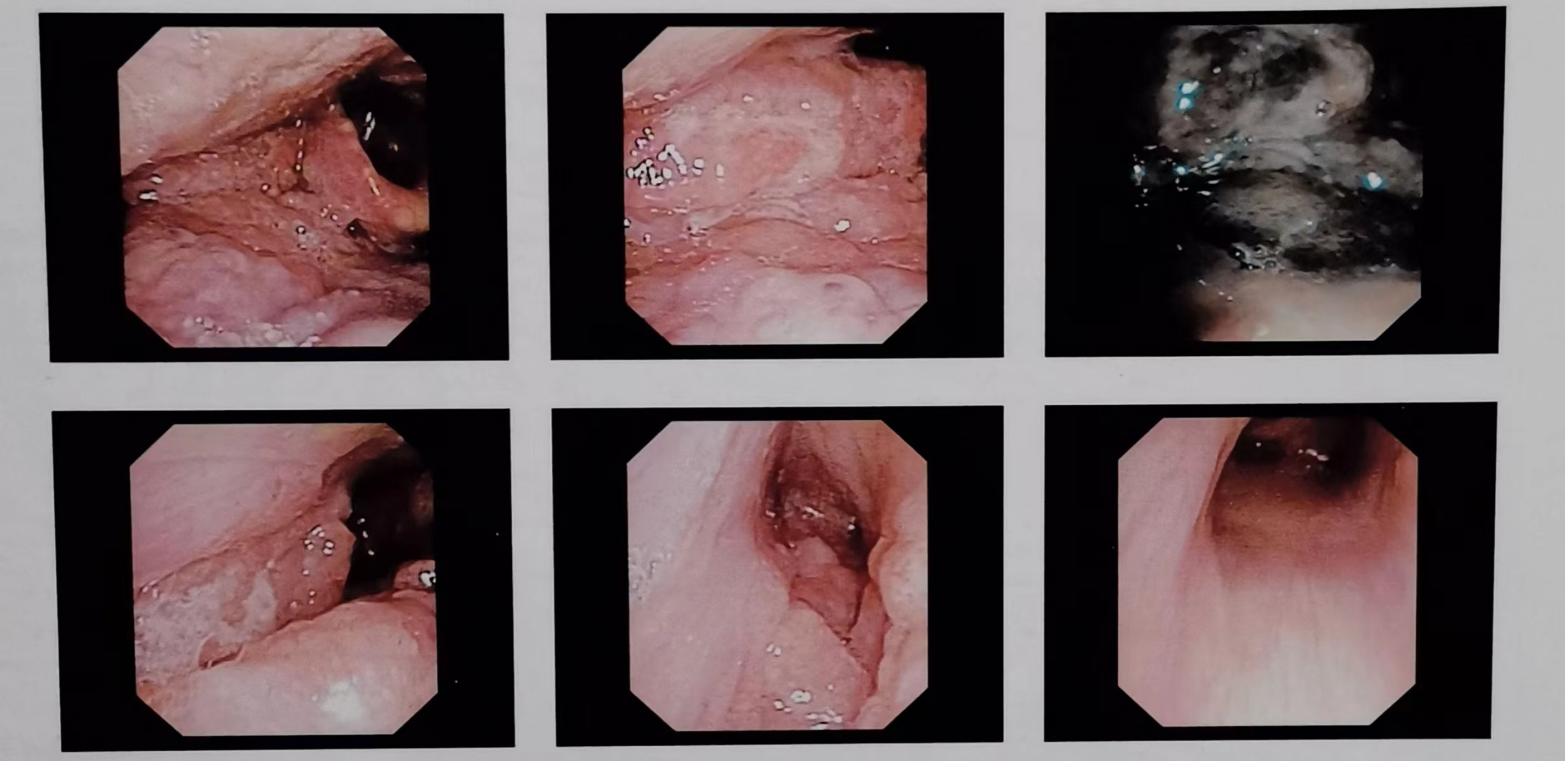
Successive laryngoscopy images showed uneven masses on the right base of the tongue, the pharyngeal side, the lingual surface of the epiglottis, the lingual epiglottis folds, the floor of the right piriform fossa, and the lateral side of the right fissure. The bilateral vocal cords were smooth and the movement was good, the bilateral fissures were well moved, and the left piriform fossa was clean.

**Figure 5:**
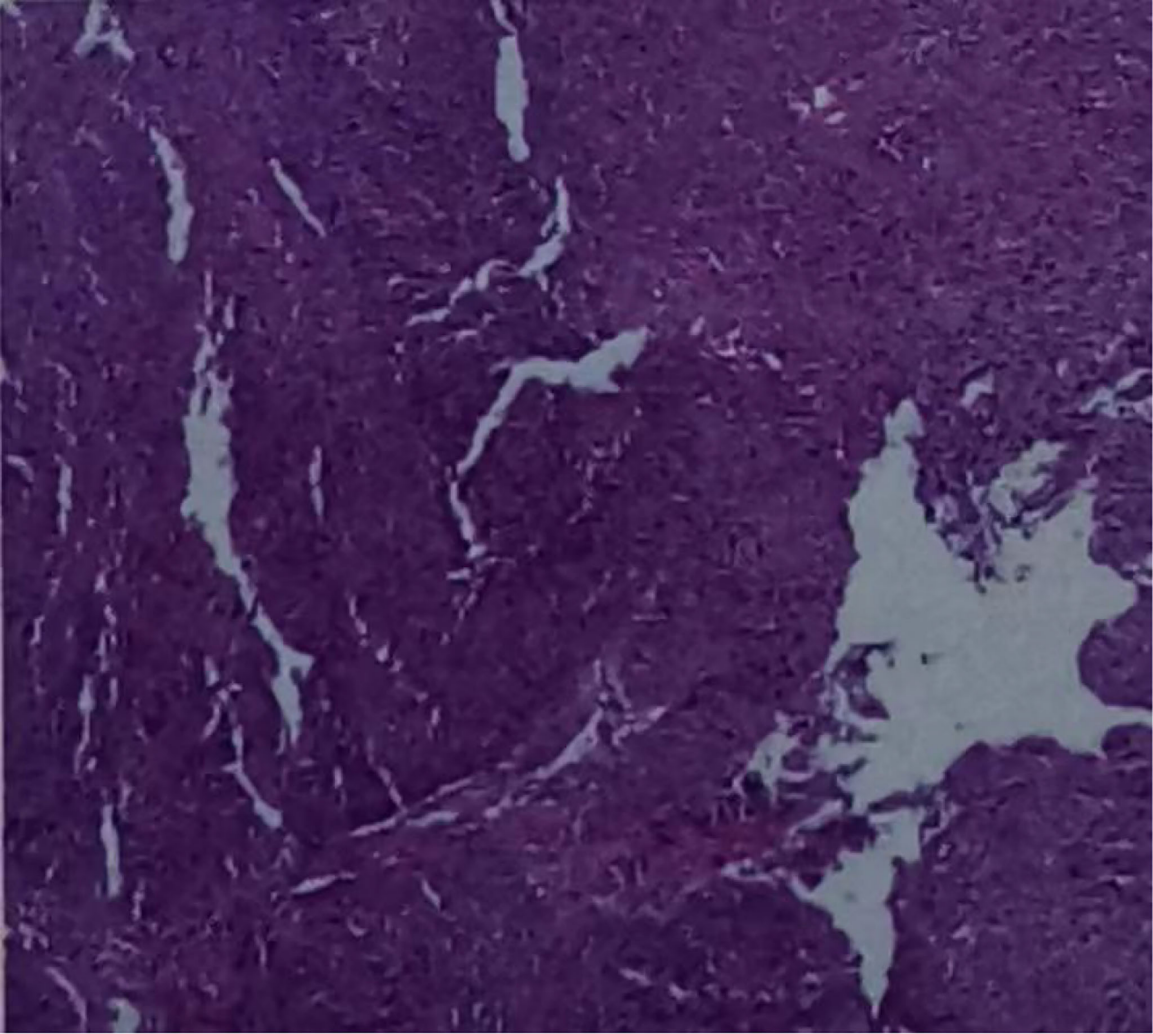
Hematoxylin-eosin stained section of hypopharyngeal invasive squamous cell carcinoma. (On July 21, 2020, a tissue biopsy specimen was taken from the right hypopharynx under laryngoscope.).

**Figure 6:**
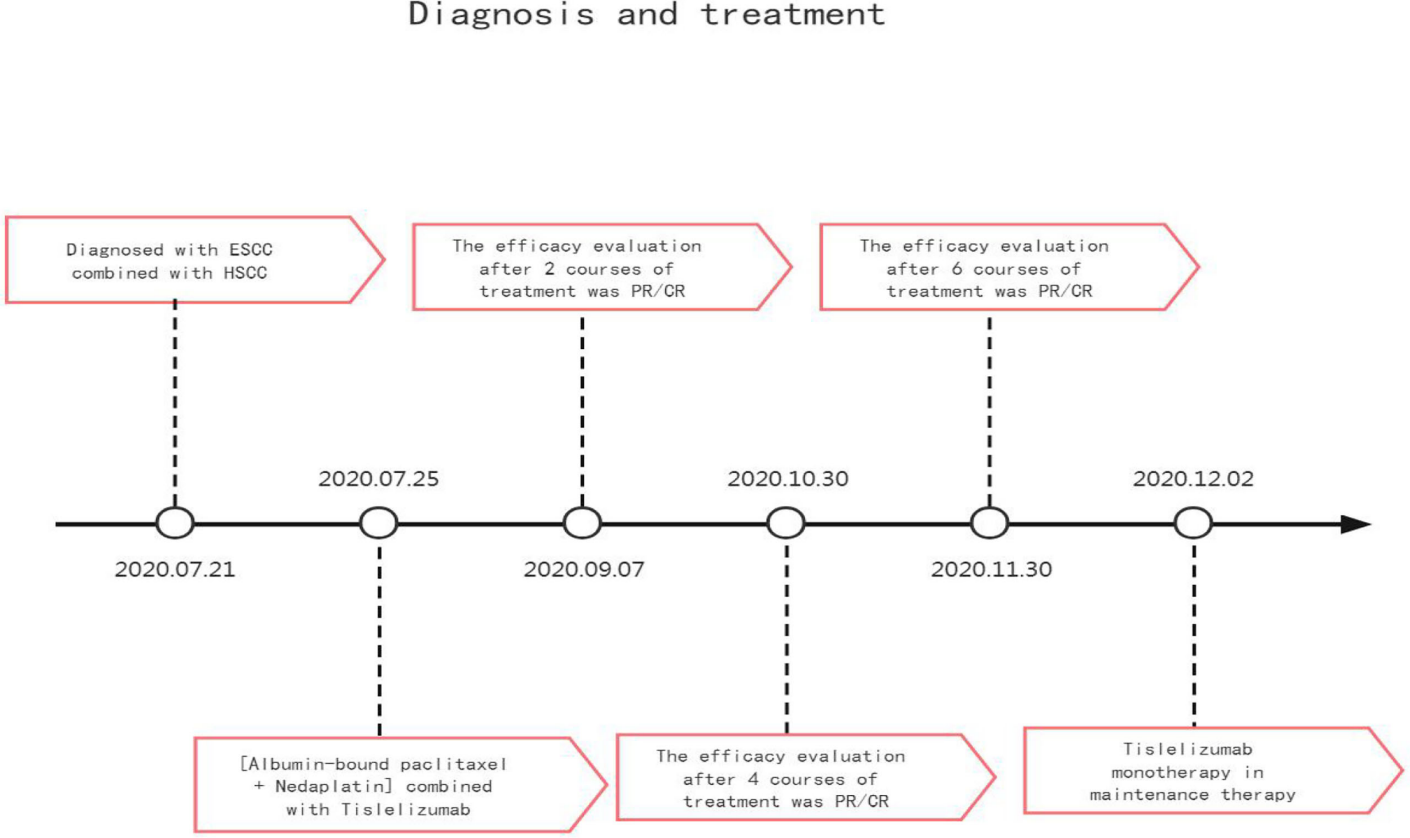
Time flow chart of the diagnosis and treatment process of this patient.

**Figure 7:**
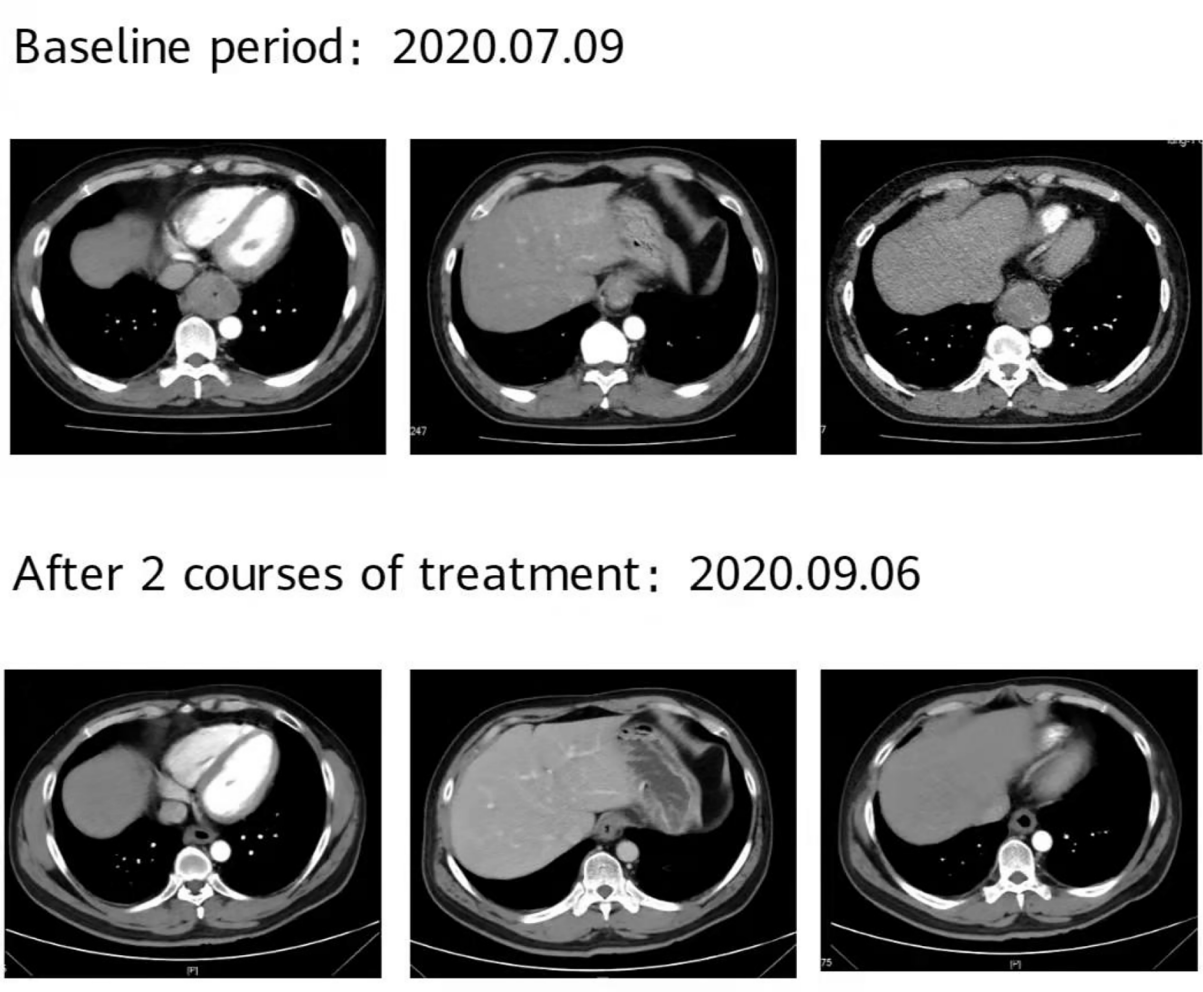
The patient’s imaging examination efficacy evaluation was based on the Response Evaluation Criteria in Solid Tumours 1.1. After 2 courses of treatment, re-examination of the second-phase enhanced CT of the lungs showed that the esophageal lesions were reduced compared with the baseline, and the curative effect was evaluated as partial remission (PR).

**Figure 8:**
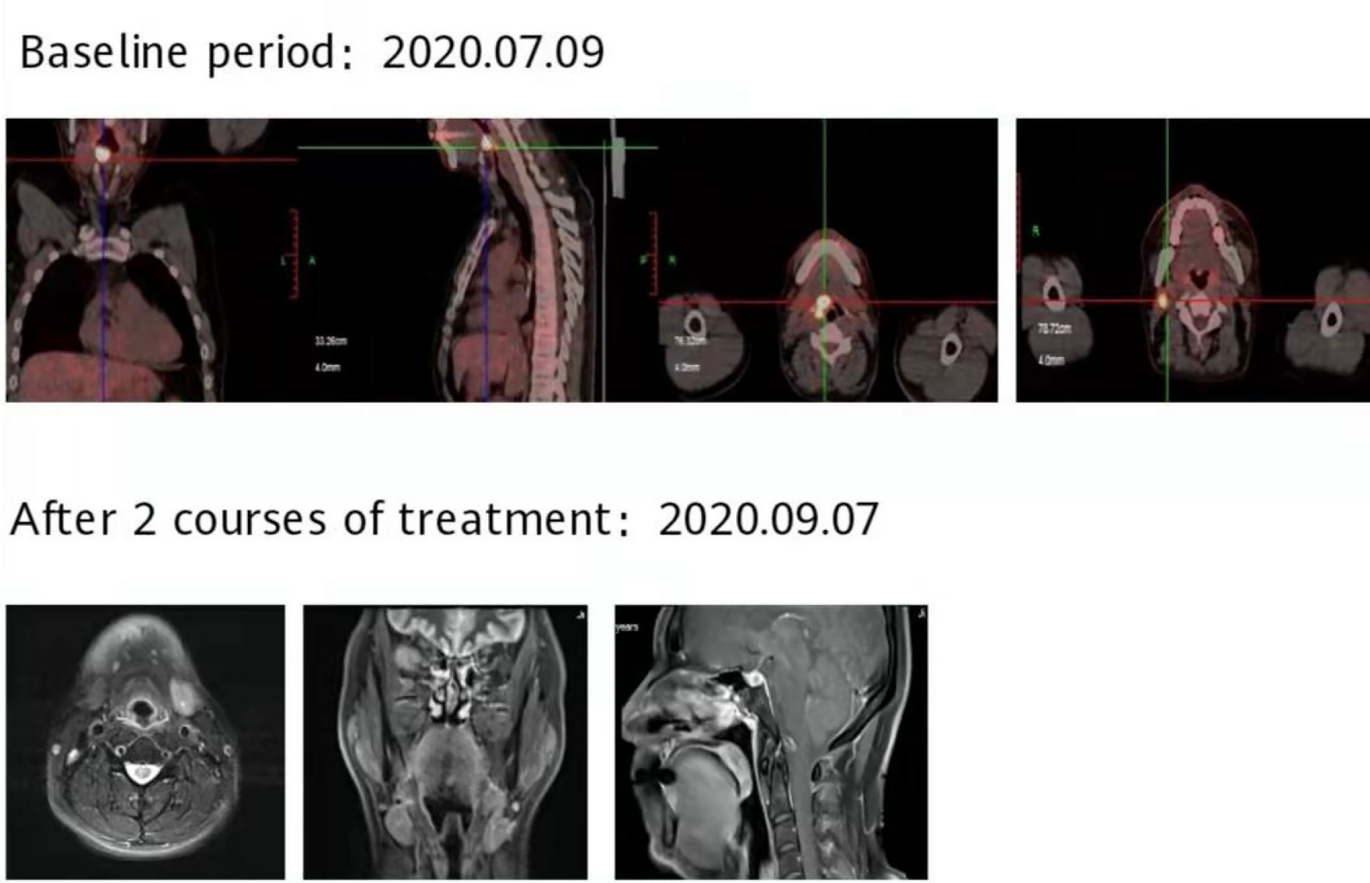
After 2 courses of treatment, no lesions were found in the MRI of the throat, and the curative effect was evaluated as complete response (CR).

**Figure 9:**
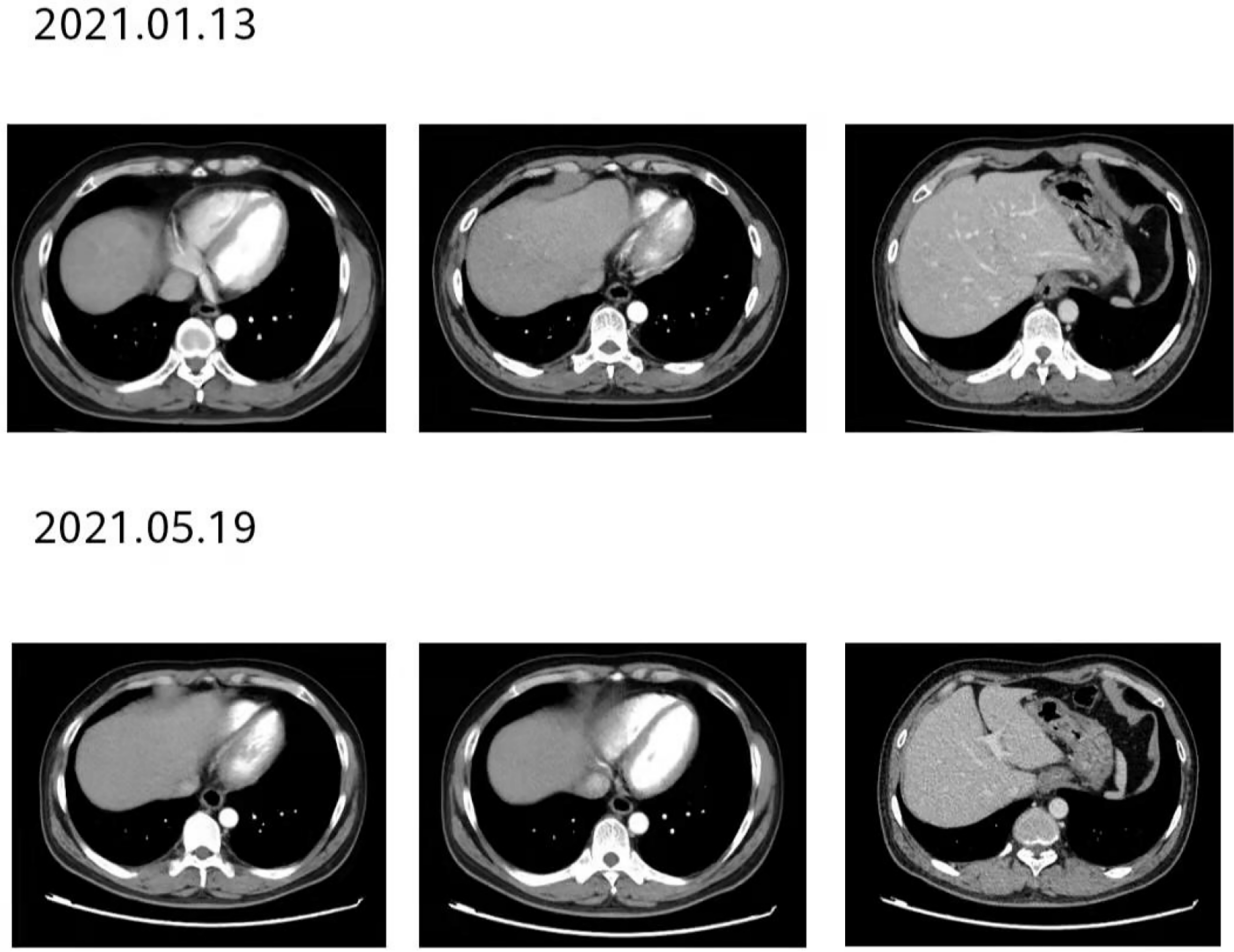
After 12 courses of treatment, re-examination of the second-stage enhanced CT of the lungs showed that the esophageal lesions were still smaller than before. Compared with that after 8 courses of treatment, the curative effect was evaluated as maintaining PR.

**Figure 10:**
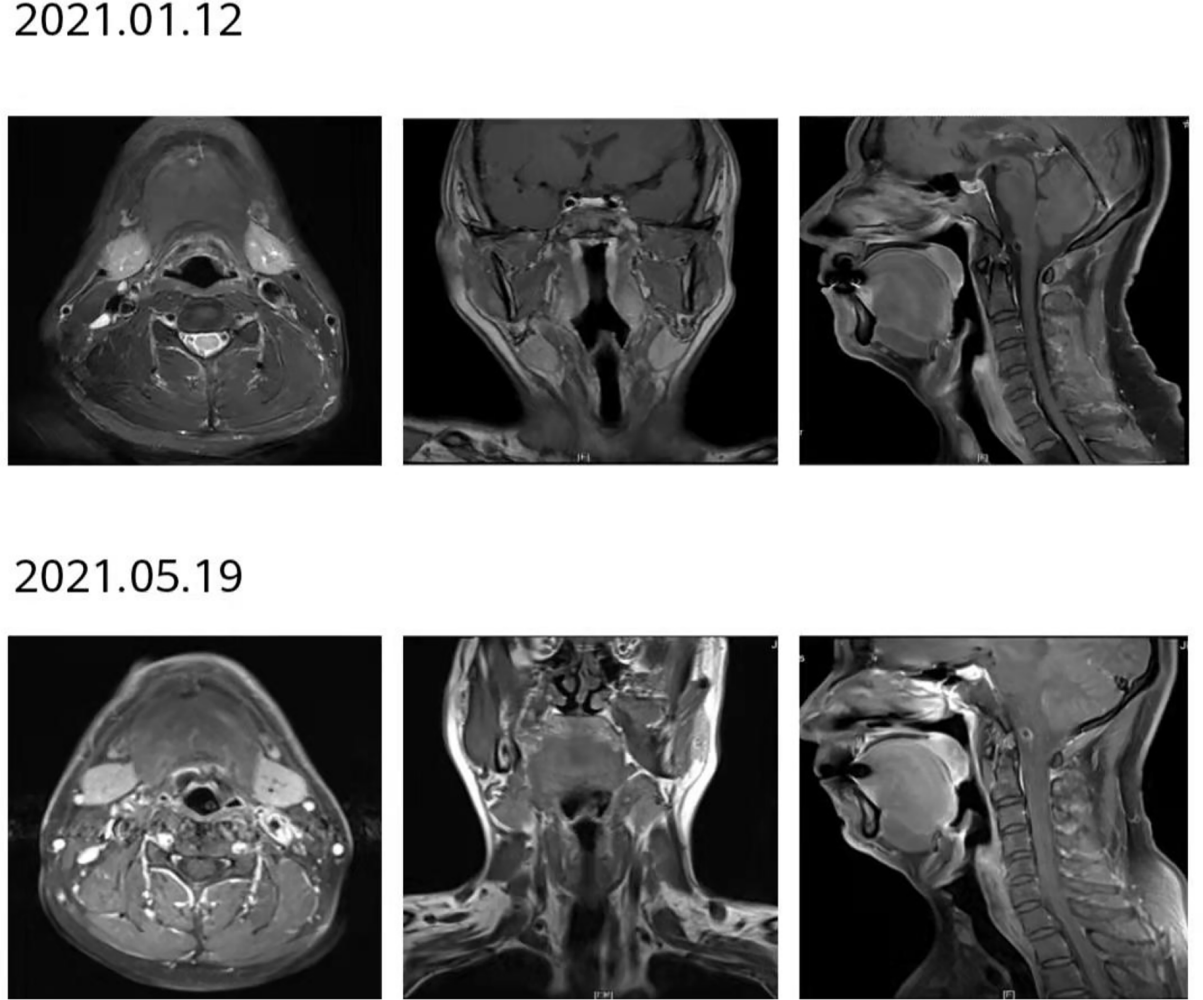
After 12 courses of treatment, no lesions were found in the reexamination of throat MRI. Compared with that after 8 courses of treatment, the curative effect was evaluated as maintaining CR.

The patient, male, 47 years old, visited our hospital on July 10, 2020 due to “choking sensation after eating”, and gastroscopy revealed an irregular ulcerated mass of about 3.0x4.0cm in size with a dyke-like elevation of the surrounding mucosa (**Figure 1**). The pathological findings of gastroscopy suggested that a low-differentiated squamous cell carcinoma in the esophagus (ESCC) (**Figure 2**), and positron emission tomography/computed tomography (PET/CT) examination for systemic evaluation suggested that the right hypopharynx had a thick wall with increased metabolism, which was considered hypopharyngeal carcinoma, and a limited hypermetabolic foci in the esophageal wall at the level of thoracic 6-10 vertebrae, which was considered esophageal carcinoma, infiltrated to the plasma membrane layer and involved the gastric cardia (**Figure 3**). Further laryngoscopy was performed to find a mass in the right hypopharynx and tongue root (**Figure 4**), the pathological findings were reported: hypopharyngeal invasive squamous cell carcinoma, and its immunohistochemical results showed: Ki-67(+30%), P40(+), CK5/6(+), P63(+) (**Figure 5**). Pathological staging was defined according to the 8th edition of the American Joint Committee on Cancer (AJCC) TNM staging system, combined with clinical, imaging and pathological diagnosis results, the patient was clinically diagnosed as lower esophageal squamous cell carcinoma (cT3N2M0 stage IIIB)、hypopharyngeal squamous cell carcinoma (HSCC) (cT1N1M0 stage III),and after multidisciplinary team (MDT) consultation, the patient did not meet the indications for surgical treatment and was recommended for systemic treatment in internal medicine, so he was given [Albumin-bound paclitaxel + Nedaplatin] combined with Tislelizumab immunotherapy starting on July 25, 2020 (the treatment flow chart is shown in **Figure 6**), and after 2 courses of treatment, he felt better choking sensation in eating, and the second-stage enhanced CT of the lung showed that the esophageal lesion was smaller than before, and the response was assessed as partial remission (PR) according to the Response Evaluation Criteria in Solid Tumours 1.1 (**Figure 7**), and no lesion was seen in the MRI of the throat on review, and the efficacy was evaluated as complete response (CR) (**Figure 8**). After 4 and 6 courses, the efficacy was assessed as maintenance PR and maintenance CR, respectively. After completing 6 courses of chemotherapy combined with immunotherapy, the patient started [Tislelizumab] single-agent immune maintenance therapy on December 02, 2020, and the efficacy was still assessed as maintenance PR and maintenance CR, respectively, after 12 courses (**Figures 9**, **10**). 13 courses of immune single-agent maintenance therapy showed no disease progression after regular review, and the lesions were still in continuous remission.

## Introduction

As the seventh most common cancer in the world and the sixth leading cause of cancer-related death, esophageal cancer has a high incidence rate in China, with more than 90% of pathological types being squamous carcinoma, and the 5-year survival rate is less than 5% for patients with inoperable esophageal cancer (1). The incidence of hypopharyngeal cancer is less than 3% among head and neck tumors, but most patients are already at advanced stage when diagnosed, and nearly 50% of them have recurrence within one year after diagnosis, with low 5-year survival rate and poor prognosis (2). Several studies have shown that patients with head and neck squamous carcinoma have a relatively high risk of death when combined with a second primary tumor, which commonly occurs in the esophagus and lung (3), and hypopharyngeal carcinoma is more likely to be combined with a second primary tumor esophageal cancer than other types of head and neck tumors, with an incidence of about 10%-50%, especially in Asian populations, but with a 5-year survival rate of only 9%-11% even after aggressive treatment (4, 5). Risk factors such as long-term smoking, alcohol consumption, betel nut chewing and chronic mucosal irritation have been shown to cause extensive carcinogenesis of the hypopharynx and esophagus (6), which may be closely related to the fact that the upper respiratory tract and upper gastrointestinal tract have similar tissue types. There is no standard effective treatment for this double primary cancer, so early and definitive diagnosis and the choice of effective treatment strategies are of paramount importance.

## Discussion

With the continuous breakthrough of anti-tumor efficacy of programmed death ligand 1 (PD-1)/programmed death ligand 1 (PD-L1) immune checkpoint inhibitors in various cancer types, the treatment of esophageal cancer and head and neck squamous carcinoma has also opened a new era of immunotherapy in recent years. Early-stage esophageal cancer is mostly treated by surgical resection, but most patients have already developed distant metastases at the time of primary treatment and lost the opportunity of surgery. At present, the first-line standard treatment is still based on fluorouracil combined with platinum-based chemotherapy, which has low overall efficiency and is prone to drug resistance after first-line chemotherapy and leads to disease progression. The publication of the KEYNOTE-181 and ATTRACTION-3 studies suggested that after failure of first-line chemotherapy in patients with esophageal squamous cancer, second-line immunotherapy has a significant overall survival (OS) benefit over chemotherapy, and based on this, the United States Food and Drug Administration (FDA) approved PD-1 blockade therapy pembrolizumab and nivolumab for second-line treatment of advanced esophageal squamous cancer (7, 8).Given the clear survival benefit achieved with PD-1 in second-line treatment of esophageal squamous carcinoma, the subsequent KEYNOTE-590 study of PD-1 inhibitor pembrolizumab in combination with chemotherapy versus placebo in combination with chemotherapy in first-line use in advanced esophageal squamous carcinoma also confirmed a clear OS benefit of immunotherapy (9), suggesting that PD-1 in combination with chemotherapy is expected to be the first-line standard of care. Similarly, in head and neck squamous carcinoma, KEYNOTE-048 (10), and the subsequent CheckMate 141 and KEYNOTE-040 studies have demonstrated a significant OS benefit of second-line application of the immune checkpoint inhibitor PD-1 (either pembrolizumab or nivolumab) over standard therapy in metastatic or recurrent head and neck squamous carcinoma (11). The publication of the above study data accelerates the FDA’s approval of PD-1 for the first-line treatment of metastatic or recurrent head and neck squamous cancer. Since there is no standard effective treatment option for esophageal squamous carcinoma combined with hypopharyngeal squamous carcinoma, we chose immunotherapy combined with chemotherapy as the first-line treatment option for this patient based on the good survival benefit results obtained from the above-mentioned clinical studies (**Table 1**).

**Table 1:** The latest treatment progress of esophageal squamous cell carcinoma and head and neck squamous cell carcinoma.

Esophageal squamous carcinoma	KEYNOTE-181	ATTRACTION-3	KEYNOTE-590
First-Line	–	–	Immunotherapy+Chemotherapy vs Placebo+Chemotherapy
Second-line	Immunotherapy vs Chemotherapy	Immunotherapy vs Chemotherapy	–
PD-1/PD-L1	Pembrolizumab	Nivolumab	Pembrolizumab
Total population OS(HR, 95%CI)	mOS:8.2 months vs 7.1 months (0.78,0.63-0.96, P=0.0095)	mOS:10.9 months vs 8.4 months (0.77,0.62-0.96, P=0.019)	12.6 months vs 9.8 months (0.72,0.60-0.88, P=0.0006)
PD-L1≥10% population mOS(HR, 95%CI)	9.3 months vs 6.7 months (0.69,0.52-0.93, P=0.0074)	–	13.9 months vs 8.8 months (0.57,0.43-0.75, P<0.0001)
**Squamous carcinoma of the head and neck**	**KEYNOTE-048**	**CheckMate 141**	**KEYNOTE-040**
First-Line	–	–	–
Second-line	Immunotherapy vs Targeted Therapy +Chemotherapy	Immunotherapy vs Chemotherapy	Immunotherapy vs Standard -chemotherapy
PD-1	Pembrolizumab	Nivolumab	Nivolumab
Total population mOS(HR, 95%CI)	PD-L1≥20%, 14.9 months vs 10.7 months (0.61,0.45-0.83, P=0.0007)	7.7 months vs 5.1 months (0.68,0.54-0.86)	8.4 months vs 6.9 months (0.80,0.65-0.98, P=0.0161)

The patient was diagnosed with esophageal squamous carcinoma combined with hypopharyngeal squamous carcinoma after the completion of gastroscopy, PET-CT and laryngoscopic biopsy,therefore, for patients with esophageal cancer or hypopharyngeal cancer, which are prone to the combination of second primary cancer, it is crucial to complete systemic examinations such as gastroscopy and otorhinolaryngoscopy during the initial treatment to clarify whether there is the combination of other systemic tumors at the same time. He could not be treated by surgical resection after MDT consultation, considering that the pathological type of squamous cell carcinoma is less likely to have gene mutation, and combined with the actual economic situation of the patient, after communicating with the patient and his family, he said that he refused to conduct further large-sample genetic testing, so we gave PD-1 inhibitor Tislelizumab in combination with [albumin-bound paclitaxel + Nedaplatin],an immunotherapy combined with chemotherapy as the first line of treatment.

As we all know, albumin-bound paclitaxel, as the third generation of paclitaxel chemotherapy, is a new type of solvent-free paclitaxel, which does not require pretreatment with glucocorticoids or antihistamines before administration, and has not only significantly improved clinical efficacy and safety tolerance compared with traditional solvent-based paclitaxel, but also has highly effective and low-toxic antitumor effects (12, 13). Meanwhile, one study found that albumin-bound paclitaxel showed good efficacy and safety in esophageal cancer and squamous cell carcinoma pathological types of head and neck tumors (14). Nedaplatin, a modified second-generation platinum-based chemotherapeutic agent, is a derivative of cisplatin and has similar antitumor activity to cisplatin, but its water solubility is 10 times that of cisplatin, and its nephrotoxicity and gastrointestinal toxicity are significantly lower than those of cisplatin (15), has been widely used in patients who cannot tolerate cisplatin and carboplatin in lung, esophageal and head and neck cancers (16). Clinical studies have shown that paclitaxel combined with nedaplatin has an ORR of 41.7%-47.7% in advanced esophageal cancer, with comparable clinical efficacy compared to conventional cisplatin chemotherapy regimens, but with better tolerability and lower toxicity (17). Of course, the chemotherapy regimen of albumin paclitaxel combined with nedaplatin has certain toxic side effects, including hematologic toxicity - thrombocytopenia, and non-hematologic toxicity - peripheral neuropathy, but the incidence of these serious adverse events is not high, and for this patient, the above-mentioned serious adverse events did not occur during the treatment.

Tislelizumab, as a domestic humanized IgG4 anti-PD-1 antibody with innovative structural modification, has a unique mechanism of action that allows it to retain the maximum number and function of effector T cells, and to specifically bind PD-1 and block the binding of PD-1 and PD-L1/PD-L2 to a greater extent, with high affinity and stronger anti-tumor activity (18). Studies have found that tumor masses are active producers of immunosuppressive substances, which can suppress the systemic immune response. Therefore, treatments that can shrink tumor tissue, such as chemotherapy, may have an impact on the overall anti-tumor immune response and its efficacy. Beneficial effects, and reduce the number and function of immunosuppressive cells in the tumor microenvironment, thus laying a good foundation for immunotherapy (19). At the same time, some studies suggest that chemotherapy can stimulate anti-tumor immune response by directly or indirectly targeting cancer cells or immune cell subsets, as well as altering systemic immune regulation, with the aim of transforming immune “cold” tumors into immune “hot” tumors, enhancing the immunogenicity of tumors and making them respond better to immune checkpoint inhibitors (20), all of the above indicate that chemotherapy combined with immunotherapy has a certain synergistic anti-tumor effect.

Although tumor mutational burden (TMB) and PD-L1 expression were not detected, this patient had a long history of heavy smoking. According to research reports, long-term exposure to tobacco environment is one of the reasons for the increase in TMB, and multiple clinical studies have shown that high expression of TMB is considered to be one of the biomarkers for good prognosis in immunotherapy, studies have found that patients with higher TMB have longer survival and higher response rates (21), which may be one of the reasons for the durable benefit of immunotherapy. In recent years, with the in-depth research on the mechanism of tumorigenesis, it has been found that the occurrence and development of tumors are closely related to chronic inflammation, and the inflammatory response can promote the development of tumors (22). Absolute neutrophil count (ANC), C-reactive protein (CRP) and neutrophil-to-lymphocyte ratio (NLR) can all reflect inflammation in the body, among them, the NLR indicator is more sensitive (23). Lactic acid dehydrogenase (LDH) is an important enzyme in energy metabolism and exists in the cytoplasm of all tissue cells, when cells are damaged, they can be released into the blood, resulting in an increase in LDH in peripheral blood. Studies have shown that serum LDH levels are generally elevated in cancer patients, and the increase in serum LDH levels after treatment often indicates a poor prognosis for patients (24). The results of multiple studies have shown that these peripheral blood biomarkers are related to the efficacy of immunotherapy and the survival prognosis of patients, low levels of ANC, NLR, and LDH before treatment or decreased from baseline after 2 courses of treatment are thought to be associated with good patient outcomes after immunotherapy (25–30). In this case, these three indicators were all at the lower limit of the normal range before treatment, and there was no increase after 2 courses of treatment, the combined existence of these three favorable factors may be closely related to the good prognosis of this case. Although there was no significant change in hematological tumor markers before and after treatment, the tumor markers were within the normal range and did not increase after repeated re-examinations after treatment, indicating that the treatment plan has a durable and effective response. At the same time, the patient was young and healthy in the past, with a performance status (PS) score of 0, chemotherapy combined with immunotherapy is well tolerated, and the compliance is very good, actively cooperate with the treatment, quit smoking immediately after diagnosis, take regular medication on a regular basis, and conduct regular review for efficacy evaluation. All of the above aspects may be closely related to the sustained survival benefit of this patient after receiving treatment. It has been reported that priming of CD4+ and CD8+ T cells contributes to signaling to cytotoxic T lymphocytes and further establishes effective and durable antitumor immunity (31), unfortunately, in this patient, changes of tumor-infiltrating CD4+ and CD8+ lymphocytes were not monitored during treatment, and it is not clear whether the patient’s durable immune response is related to the increase in CD4+ and CD8+ after treatment.

Although the mechanism of immunotherapy-related adverse events is not clear, studies believe that this is an autoimmune reaction after blocking the normal immune regulation pathway, common adverse reactions are endocrine system-related adverse reactions, including immune-related thyroid toxicity, hypophysitis, adrenal insufficiency and diabetes, etc, the most common occurrence is immune-related thyroid toxicity, including thyrotoxicosis, hypothyroidism and thyroiditis. Hypothyroidism generally occurs around 10-20 weeks after receiving immunotherapy and is defined as high thyroid stimulating hormone(TSH) with low Free T4 (FT4) or total T3 (TT3) (32). In this patient, an immune-related adverse reaction event-hypothyroidism inevitably occurred after treatment, but it appeared later. When the five items of thyroid function were reviewed about 37 weeks after treatment, it was found that the TSH was 74.082uIU/ml (the normal range is 0.35-4.94 uIU/ml), FT4<5.15pmol/L (the normal range is 9.01-19.05 pmol/L), after consultation with the endocrinology department, a diagnosis of hypothyroidism is recommended. It is recommended to take Euthyrox(levothyroxine sodium tablets) regularly, later, regular review of thyroid function was within the normal range, but the patient may need to take Euthyrox for life.

In this case, after 2 courses of immuno-combination chemotherapy, the tumor in the esophagus shrank significantly compared with before, and the efficacy was assessed as PR, and the tumor in the hypopharynx disappeared, and the efficacy was assessed as CR, and the efficacy was assessed as maintenance PR/maintenance CR after 6 courses, and the efficacy was assessed as maintenance PR/maintenance CR after 13 courses of immuno-monotherapy maintenance treatment, and no disease progression was seen at regular review, and the PFS exceeded 15 months. The patient has preserved pharyngeal function and normal feeding, although immune-related adverse reactions occurred after the medication, the symptomatic medication was well controlled and did not affect daily life, and no other adverse reactions occurred, which ensured the survival benefit while ensuring the quality of life. This suggests that first-line use of immune-combination chemotherapy may have a sustained survival benefit in patients with esophageal squamous carcinoma combined with hypopharyngeal squamous carcinoma that is safe and tolerable. However, there is still a lack of case reports and relevant clinical studies on the treatment effect of such patients with dual primary cancers. Therefore, a large number of actual clinical studies are still needed to explore and discover better treatment decisions and bring longer and more favorable survival benefits for such patients.

## Data Availability Statement

The raw data supporting the conclusions of this article will be made available by the authors, without undue reservation.

## Author Contributions

YY contributed to write the manuscript. XL, WS, JL contributed to perform efficacy assessment. NY, XJ contributed collect clinical information from patients. XC contributed to manuscript revision. All authors contributed to the article and approved the submitted version.

## Conflict of Interest

The authors declare that the research was conducted in the absence of any commercial or financial relationships that could be construed as a potential conflict of interest.

## Publisher’s Note

All claims expressed in this article are solely those of the authors and do not necessarily represent those of their affiliated organizations, or those of the publisher, the editors and the reviewers. Any product that may be evaluated in this article, or claim that may be made by its manufacturer, is not guaranteed or endorsed by the publisher.
